# GTExVisualizer: a web platform for supporting ageing studies

**DOI:** 10.1093/bioinformatics/btad303

**Published:** 2023-05-08

**Authors:** Pietro Hiram Guzzi, Ugo Lomoio, Pierangelo Veltri

**Affiliations:** Department of Surgical and Medical Sciences, Magna Graecia University of Catanzaro, Viale Europa, Catanzaro 88100, Italy; Data Analytics Research Center, University of Catanzaro, Catanzaro, Italy; Department of Surgical and Medical Sciences, Magna Graecia University of Catanzaro, Viale Europa, Catanzaro 88100, Italy; DIMES, University of Calabria, Via Pietro Bucci, Rende, Italy

## Abstract

**Motivation:**

Studying ageing effects on molecules is an important new topic for life science. To perform such studies, the need for data, models, algorithms, and tools arises to elucidate molecular mechanisms. GTEx (standing for Genotype-Tissue Expression) portal is a web-based data source allowing to retrieve patients’ transcriptomics data annotated with tissues, gender, and age information. It represents the more complete data sources for ageing effects studies. Nevertheless, it lacks functionalities to query data at the sex/age level, as well as tools for protein interaction studies, thereby limiting ageing studies. As a result, users need to download query results to proceed to further analysis, such as retrieving the expression of a given gene on different age (or sex) classes in many tissues.

**Results:**

We present the GTExVisualizer, a platform to query and analyse GTEx data. This tool contains a web interface able to: (i) graphically represent and study query results; (ii) analyse genes using sex/age expression patterns, also integrated with network-based modules; and (iii) report results as plot-based representation as well as (gene) networks. Finally, it allows the user to obtain basic statistics which evidence differences in gene expression among sex/age groups.

**Conclusion:**

The GTExVisualizer novelty consists in providing a tool for studying ageing/sex-related effects on molecular processes.

**Availability and implementation:**

GTExVisualizer is available at: http://gtexvisualizer.herokuapp.com. The source code and data are available at: https://github.com/UgoLomoio/gtex_visualizer.

## 1 Introduction

Becoming older or ageing represents the accumulation of phenotype changes in humans over time (Booth and Brunet 2016). Ageing may be explained as a set of *ageing clocks*, identifying a subset of modifications for molecules which could be considered age evolution predictors. Observing these changes might determine a measure of *biological age* slightly different from *anagraphic age*, thus providing information about the risk of disease development. The study of ageing processes at the molecular level requires the integration and analysis of multiomics data, often available in public databases (Fabris et al. 2017), together with age and sex information. GTEx portal (Lonsdale et al. 2013) allows to access human multiomics data. It contains gene samples of anonymous donors annotated with metadata related to: tissue of provenance, sex, and age (Mercatelli et al. 2021). Nevertheless, due to limited query expression language, GTEx does not allow the user to perform data analysis for molecular ageing studies. For instance, data cannot be grouped or filtered by using age and sex, or partial results cannot be integrated with existing protein interaction databases by using the same portal. When researchers want to analyse data at the sex/age level, they have to write a script file to download data and then locally analyse them. This is a significant limitation especially for non-experts. Moreover, reconstructing and studying ageing processes at the network level is lacking, hence the need to gather information from different sources to build omics networks, e.g. by gathering data from STRING-DB protein-to-protein interaction database (Szklarczyk et al. 2021).

We present GTExVisualizer, a framework able to: (i) gather data from GTEx data portal and from STRING-DB interaction database and (ii) integrate and analyse data by using a simple web interface. The user can query and analyse GTEx data by grouping samples by tissue, sex, and age adding filters and defining plots. Users can also retrieve protein interaction networks from STRING-DB by using the genes filtered by age and sex values. To the best of our knowledge, GTExVisualizer is the only web-based tool allowing the user to query and analyse GTEx data using sex and age information and thus supporting molecular ageing studies. Moreover, we considered GTEx source since the existing ones do not present age and sex-related information. Supplementary Table S1 reports on the available existing resources comparing them with GTExVisualizer.

## 2 Results

**Figure 1.**
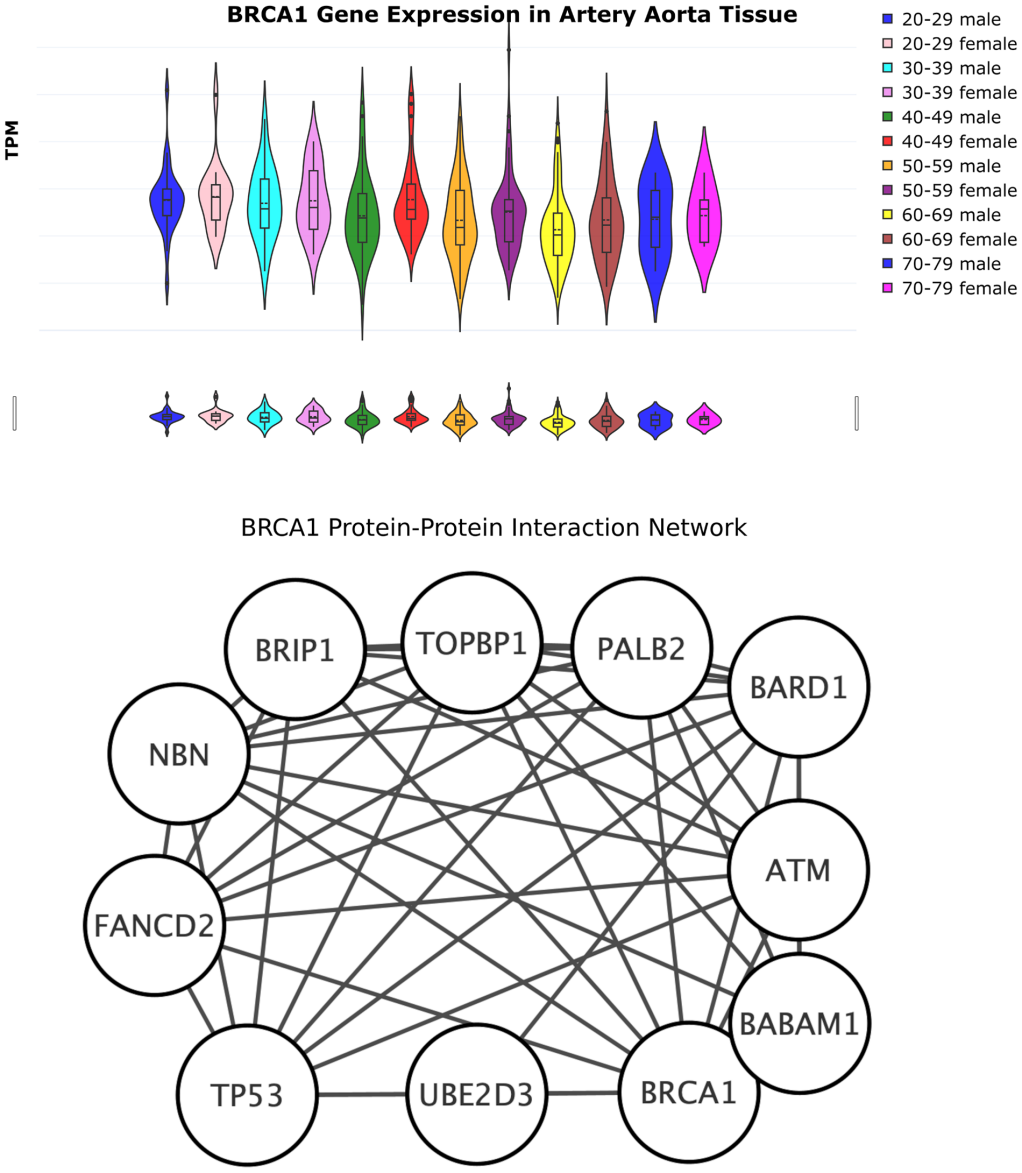
BRCA1 expression and the related protein interaction network in (artery) aorta tissue when the age/sex filter is selected. Gene expression is measured as Transcript per Kilobase Million TPM.

The GTExVisualizer has been implemented by using Python programming language, and it is accessible at gtexvisualizer.herokuapp.com. Data can be queried by using a gene name, (e.g. BRCA1) and a human tissue (e.g. aortha) as filters. Then, age interval (e.g. 20–29 years old), sex (male or female) and a combination of both are used to plot and study results. Results are visualised by using *violin* plots and can be related to protein interaction networks of the selected genes. Figure 1 shows an example of using GTExVisualizer filter, sex–age plot, and protein interaction network associated with the gene used in the filter phase (BRCA1 in the example).

The upper part of the figure shows a violin plot of the expression data. Data are grouped for each age group as described in the legend. Users may download both plots and expression data for further analysis. The GTExVisualizer results also contain statistical analysis such as: (i) one-way analysis of variance (ANOVA) and one-way ANOVA on ranks to assess the existence of differences between classes and (ii) Shapiro–Wilk to test the normal distribution of data. In this way, the user may easily infer the presence of a significant difference between age/sex groups. For instance, modification of gene expression can be associated with age and/or sex. These tests enable us to answer important queries such as: (i) does the level of the BRCA1 gene change with age in the selected tissue?; (ii) does the level of the BRCA1 gene have different behaviour in males/females with age. Additional plots such as sample donor description are also provided in the tool. The whole set of plotting functions is described in the GTExVisualizer online user manual.

## 3 Conclusion

The presented framework allows the user to perform molecular ageing studies on human transcriptomic data contained in the GTEx portal, exploiting sex, age, and tissue information. The GTExVisualizer also enables to relate the study results into protein-to-protein interaction networks extracted from STRING-DB database. The results are then plotted providing additional information for ageing studies. The proposed framework currently uses only GTEx data. This is due to the fact that metadata of interest for molecular ageing studies are not available in other portals. Future directions may involve the design of a general purpose platform integrating sex/age-related data gathered from available data sources (i.e. raw data), as well as a general purpose framewor release allowing to gather and analyse user defined data.

## Supplementary Material

btad303_Supplementary_Data
