# Correction to: GTExVisualizer: a web platform for supporting ageing studies

**DOI:** 10.1093/bioinformatics/btae094

**Published:** 2024-02-22

This is a correction to: Pietro Hiram Guzzi, Ugo Lomoio, Pierangelo Veltri, GTExVisualizer: a web platform for supporting ageing studies, *Bioinformatics*, Volume 39, Issue 5, May 2023, btad303, https://doi.org/10.1093/bioinformatics/btad303.

In the originally published version of this manuscript, there was an error in the spelling of Pietro Hiram Guzzi’s name.

This error has been corrected.

