# Supplementary material for: GTExVisualizer: a web platform for supporting ageing studies
Source: Bioinformatics. 2023 May 8;39(5):btad303. doi: 10.1093/bioinformatics/btad303 (PMC10196670; doi:10.1093/bioinformatics/btad303)
Supplement: btad303_Supplementary_Data [file btad303_supplementary_data.pdf]

Supplementary Table 1

**TOOL Name**	**Data Sample**	**Data granularity: Tissue information**	**Data granularity: Sex information**	**Data granularity: Age information**	**Network Database instances**	**Multiple datasets**	**Automatic Statistical Analysis**	**Visualization and plot representation tools**
GTExVisualizer	Patients	available	available	available	yes accessing to STRING-DB	no, access only to GTEx portal dataset	yes	Yes (Violin Plots)
XenaBrowser	Patient	not available	not available	not available	not present	yes, it contains to multiple datasources	no	no
Expression Atlas	Patient	available	available	not available	not present	yes, it contains to multiple datasources	no	no
GenAge	Cell	not available	not available	available (partially)	not present	no	no	no
Longevitiy Map	human cell	not available	not available	available (partially)	not present	yes Multiomic	no	Yes
Aging Atlas	Cell	not available	not available	available	yes	yes, Multiomics	No	Yes

Table 1: Table reports information regarding data and granularity for some of the existing genomic data sources. It is possible to compare them with GTExVisualizer.

References

XenaBrowser https://xenabrowser.net/
Expression Atlas https://www.ebi.ac.uk/gxa/home
GenAge https://genomics.senescence.info/genes/index.html
Longevity Map: https://genomics.senescence.info/longevity/
Aging Atlas : https://ngdc.cncb.ac.cn/aging
